# Differential effects of frozen storage on the molecular detection of bacterial taxa that inhabit the nasopharynx

**DOI:** 10.1186/1472-6890-11-2

**Published:** 2011-01-24

**Authors:** Brenda A Kwambana, Nuredin I Mohammed, David Jeffries, Mike Barer, Richard A Adegbola, Martin Antonio

**Affiliations:** 1 Bacterial Diseases Programme, Medical Research Council Laboratories (UK), Banjul, The Gambia; 2 Department of Infection, Immunity, and Inflammation, University of Leicester, Leicester, UK; 3 Bill & Melinda Gates Foundation, Seattle, USA

## Abstract

**Background:**

Frozen storage often precedes metagenomic analysis of biological samples; however, the freezing process can have adverse effects on microbial composition. The effect of freezing on the detection of bacteria inhabiting the infant nasopharynx, a major reservoir of bacterial pathogens, was investigated.

**Methods:**

16S ribosomal RNA (rRNA) gene-based terminal restriction fragment length polymorphism (T-RFLP) analysis of nasopharyngeal (NP) swabs from twelve Gambian infants was employed. NP swabs were analysed within hours of collection and then after 30 days of storage at -70°C.

**Results:**

There was substantial heterogeneity among subjects with respect to the effect of freezing on the number of operational taxonomic units (OTUs) detected. Nevertheless, the mean number of OTUs decreased after frozen storage and the relative abundance for 72% of the OTUs changed by less than 0.5% after deep frozen storage. There were differences in the odds of detection and relative abundance of OTUs matched with *Moraxella sp*., *Haemophilus sp*./*Burkholderia sp.*, and *Pseudomonas sp*. A strong interaction between sex and the effect of freezing was found, whereby there was no significant change observed for males while the mean number of OTUs significantly declined among female infants following frozen storage.

**Conclusions:**

Although frozen storage of biological samples is often necessary for archiving and logistic purposes, the potential effects on the number of taxa (composition) detected in microbial community studies are significant and should not be overlooked. Moreover, genetic factors such as sex may influence the integrity of nucleic acids during the freezing process.

## Background

Nasopharyngeal swabbing is an important diagnostic and epidemiological surveillance tool used to detect several pathogens in one of the most clinically relevant microbial reservoirs in the human body [1,2]. Although detection of microbes in nasopharyngeal (NP) swabs stored in skim milk-tryptone-glucose-glycerin (STGG) is often employed in epidemiology and diagnostic research [3], few studies have investigated the potential bias introduced during sample storage, which usually involves deep freezing at -20°C or -70°C [1,4-6]. Furthermore, these studies have focused on the effects of frozen storage on the culture-based detection of specific pathogens such as *Streptococcus pneumoniae*. However, the impact of freezing on the molecular culture-independent detection of the microbial communities inhabiting the nasopharynx is yet to be fully understood.

With the widespread application of vaccines and antibiotics against respiratory pathogens, serotype and species replacement is becoming increasingly important. The occurrence of species replacement depends on the ecological events that occur during the replenishment of the niche left vacant following vaccination or antibiotic treatment. Hence, extensive microbial ecology studies will be necessary to fully understand these events and identify microbes that play a significant role in replenishment. Furthermore, the competitive relations between bacterial types inhabiting the nasopharynx which may alter predisposition to disease will be elucidated by broad comparative community analyses. This reinforces the need to broaden the scope of upper airway microbiology studies [7-10]. However, only a small fraction of bacterial composition can be cultivated by standard laboratory techniques [11,12]. Hence, culture-dependent applications grossly underestimate species richness, relative abundance and composition, limiting their utility in the comprehensive analysis of microbial communities [13]. To address various aspects of diverse microbial communities, microbiologists have employed an extensive array of culture-independent molecular tools which are often based on PCR and target the rRNA gene cluster [14,15]. Although molecular tools avert considerable bias introduced by selective cultivation and have revolutionized our understanding of microbial ecology, they also have several limitations. Common to all PCR-based applications are several pitfalls which can occur at every stage of sample processing [13]. Bias can be introduced during sample collection and transport, sample storage, cell lysis, nucleic acid extraction, PCR amplification, and other downstream applications [13,16-20]. One of the advantages of molecular applications is that samples can be stored and analysed even after the loss of viability of microbial cells. For logistic and technical reasons, frozen storage often precedes the molecular analysis of biological samples. However, it is becoming increasingly evident that the deep freezing process can have adverse effects on microbial composition and the detection of specific bacterial strains [4,21,22].

Prior to a study characterizing the development of the infant nasopharyngeal microbiome, we set out to validate an effect of frozen storage of NP swabs on the molecular detection of bacterial taxa using 16S rDNA based-Terminal restriction fragment length polymorphism (T-RFLP). T-RFLP is a PCR based DNA fingerprinting tool that has been applied in community analyses across different ecosystems [23]. To our knowledge this is the first investigation of an effect of freezing on the 16S rRNA gene-based profiling of microbial communities of the infant nasopharynx.

## Methods

### Study site and sample collection

On the same day, a nasopharyngeal swab was collected from each of twelve Gambian infants (5 male and 7 female). Infants were recruited from the Western Region with parental informed consent as previously described by Hill et *al*., 2008 [24]. The study was approved by the Medical Research Council Scientific Coordinating Committee and Joint Gambian Government Ethics Committee. Trained field nurses used sterile calcium alginate fibre tipped swabs with aluminium shafts (Fisher brand^®^, USA), which were immediately placed in 1 mL of STGG medium, kept on ice and transported to the laboratory within 4 hours of collection as previously described. At the time of sampling six infants were 16 weeks old and six were 26 weeks old. Subject data is summarized in Table 1.

**Table 1:** Description of samples collected for this study

Subject	Sex	NP Swabs Collected	Fresh DNA Extractions	Frozen Storage	Frozen DNA Extractions	Age at Collection (Weeks)	Before Frozen Storage	95% CI (Poisson)	After Frozen Storage	95% CI (Poisson)
**1**	**M**	**1**	**2**	**30 days**	**2**	**16.9**	**12.0**	**7.7:17.9**	**14.5**	**9.7:20.8**
**2**	**M**	**1**	**2**	**30 days**	**2**	**16.6**	**3.5**	**1.4:7.2**	**11.5**	**7.3:17.3**
**3**	**F**	**1**	**2**	**30 days**	**2**	**16.4**	**8.0**	**4.6:13.0**	**1.5**	**0.3:4.4**
**4**	**F**	**1**	**2**	**30 days**	**2**	**16.1**	**13.5**	**8.9:19.6**	**0**	0:1.8*
**5**	**F**	**1**	**2**	**30 days**	**2**	**16.1**	**9.5**	**5.7:14.8**	**0**	0:1.8*
**6**	**F**	**1**	**2**	**30 days**	**2**	**16.0**	**10.0**	**6.1:15.4**	**1.0**	**0.1:3.6**
**7**	**M**	**1**	**2**	**30 days**	**2**	**26.6**	**6.5**	**3.5:11.1**	**3.5**	**1.4:7.2**
**8**	**F**	**1**	**2**	**30 days**	**2**	**26.6**	**3.5**	**1.4:7.2**	**5.5**	**2.7:9.8**
**9**	**F**	**1**	**2**	**30 days**	**2**	**26.3**	**6.5**	**3.5:11.1**	**5.0**	**2.4:9.2**
**10**	**F**	**1**	**2**	**30 days**	**2**	**25.9**	**10.0**	**6.1:15.4**	**9.0**	**5.3:14.2**
**11**	**M**	**1**	**2**	**30 days**	**2**	**26.1**	**14.0**	**9.3:20.2**	**12.0**	**7.7:17.9**
**12**	**M**	**1**	**2**	**30 days**	**2**	**26.1**	**11.5**	**7.3:17.3**	**12.0**	**7.7:17.9**

(*) one-sided, 97.5% confidence interval (CI)

### DNA Extraction

Duplicate nucleic acid extractions were carried out for each of the twelve NP swabs prior to and post freezing, giving a total of 48 purified DNA samples. 100 μL of each NP swab were extracted using the UltraClean^® ^Microbial DNA Isolation Kit (Mo-Bio, USA) following manufacturer's protocol. For direct analysis, DNA was extracted from NP swabs kept on ice within 4 hours of collection. For frozen analysis, DNA was extracted from the NP swabs frozen at -70°C for 30 days and gently thawed on ice. DNA was eluted in 50 μL of elution buffer (MD5) and stored at -20°C. Duplicate extractions were carried out for both fresh and frozen analyses to control for variability associated with the extraction process.

### PCR for T-RFLP Analysis

The PCR was optimized to produce minimal amounts of primer dimer which can interfere with T-RFLP analysis. 16S rDNA amplification yielding fragments ~700 bp was performed in 25 μL reaction volumes consisting of 2.5 μL of DNA, 1x Green GoTaq reaction buffer with 1.5 mM MgCl_2_, 1.0 U Go*Taq *Polymerase (Promega, UK), each deoxynucleoside triphosphate at 0.2 mM of each deoxynucleoside triphosphate (dNTP) (QIAGEN, UK), 0.5 μM of 6-carboxyfluorescein labelled forward primer 338F-[ 6-FAM] (5'-ACT CCT ACG GGN GGC NGC A-3') (Applied Biosystems, UK) and 0.25 μM 1046R (5'-CAC GAG CTG ACG ACA NCC ATG CAN CAC C-3'). Amplification was carried out by initial denaturation of 94°C for 1 minute, followed by 30 cycles of denaturation at 94°C for 1 minute, annealing at 58°C for 1 minute, and extension at 72°C for 2 minutes. Final extension was done at 72°C for 10 minutes. Duplicate PCR products were pooled and purified with the QIAquick PCR Purification Kit (QIAGEN, UK) and stored at -20°C. PCR products were checked by 1.5% agarose gel electrophoresis and PCR products were quantified by NanoDrop spectrophotometer. Duplicate amplifications were done for each DNA sample and pooled to reduce bias associated with PCR.

### T-RFLP Analysis

Approximately 100 ng of DNA were digested with 5U of restriction enzyme *Alu*I (NEB, UK) for 3 hours at 37°C, followed by 20 minutes at 65°C. Approximately equal amounts of PCR product from each sample were analysed to reduce the effects of fingerprinting different amounts of DNA. Digested amplicons were cleaned using SureClean (Bioline, UK) and eluted in 10 μL 10 mM Tris-Cl, pH 8.5 (QIAGEN, UK). 1 μL (~10 ng) of purified digest, 0.5 μL of GeneScan-600 Liz and 8.5 μL of Hi-Di™ formamide (Applied Biosystems, UK) were mixed followed by DNA denaturation at 95°C for 3 minutes. Fragments were separated by size by capillary electrophoresis on a 3130*xl *Genetic Analyzer (Applied Biosystems, UK) and visualized by excitation of the 6-FAM labelled attached to the 5' terminal fragment by the following protocol: Oven temperature 60°C, injection voltage 1.6 kV, injection time 15 s, running voltage 15 kV and running time 2,500 s.

### Reference nasopharyngeal bacteria library

DNA extracts of NP swabs collected from 6 infants were used as template for the amplification of 16S rRNA fragments, using unlabelled universal primers 338F and 1046R as described above. Purified 16S rDNA fragments were directly cloned using the pGEM^®^-TEasy Vector System following manufacturers protocol (Promega, UK). Putative positive colonies were selected by blue-white screening and cultured overnight in 100 μg/mL ampicillin LB broth. Inserts were amplified and sequenced using M13/pUC primers M13/pUCF (5'CCCAGTCACGACGTTGTAAAACG-3'), M13/pUCR (5' AGCGGATAACAATTTCACACAGG-3'), (Promega, UK) following manufacturers protocol. Raw sequences were edited using Lasergene SeqMan (DNASTAR, UK). *In silico *digest of sequences was carried out using WebCutter 2.0 (http://rna.lundberg.gu.se/cutter2/) and matched with corresponding T-RFs. Sequences were screened for the presence of chimeras with the Mallard chimera-checking tool prior to submission. 165 partial 16S rRNA gene nucleotide sequences were deposited onto GenBank http://www.ncbi.nlm.nih.gov/Genbank and assigned accession numbers (HM179296 to HM179460).

### T-RFLP Statistical Analysis

The electropherograms were analysed with GeneMapper^® ^Software v4.0 (Applied Biosystems). The advanced peak detection algorithm was used and fragment sizes were determined by the Local Southern method and fluorescence signal was normalized for all the samples analysed. A fixed detection threshold of 100 fluorescence units (FU) was used to reduce inclusion of noise peaks in the analysis [23,25]. Terminal restriction fragments (T-RFs) that differed by ± 1 bp in different profiles were binned in the same OTU. T-RFs between 50 bp and 600 bp were considered in the analysis to obtain 5' terminal fragments within the linear range of GS-600Liz (Applied Biosystems). Composition was calculated as the total number of distinct OTUs detected in a sample. The relative abundance of each OTU was determined by expressing the fluorescence units as a proportion of the sum of relative fluorescence signal for each sample [26]. A random effects model allowing for the within subject correlation was used to investigate the effect of freezing on composition and total RFUs per subject adjusting for age and sex. A random effects logistic regression model was used to study the association of detection of each OTU with the type of sample (frozen versus fresh) adjusting for age and sex where appropriate. Only OTUs detected in both primary and frozen samples were included and correction for multiple testing was done using Holm's method.

## Results

Duplicate NP DNA extracts from each of the twelve infants were analysed within hours of collection and after thirty days of frozen storage at -70°C in STGG. Hence, a total of 48 nucleic acid samples were analysed by 16S rDNA T-RFLP (Table 1). Terminal restriction fragments (T-RFs) were not generated for seven samples, six frozen and one fresh sample due to poor amplification. Hence, 96% and 75% of samples analysed fresh and after frozen storage were successfully analysed by T-RFLP respectively. A total of 42 distinct OTUs were detected in the NP swabs, 86%, 74%, and 62% were detected in fresh analysis only, after frozen storage only and in both analyses respectively.

**Figure 1:**
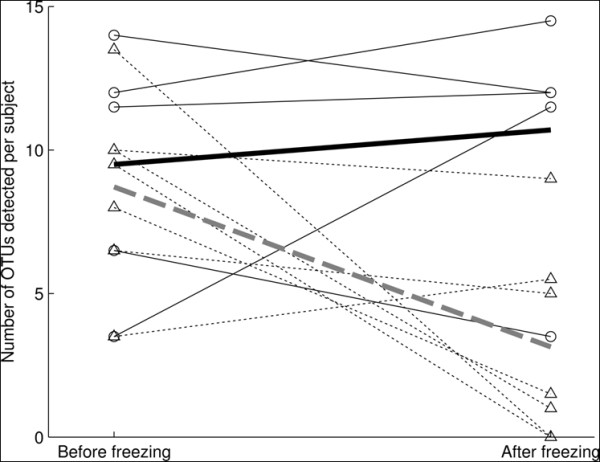
**Effect of frozen storage on the composition of bacterial OTUs found in the infant nasopharynx**. The composition (mean number of OTUs) per subject against 16S rRNA-based T-RFLP before and after freezing at -70°C dichotomized by sex. Dotted lines represent females, and the bold dotted line shows the mean change. The difference in composition pre and post freezing was significant for female (p = 0.0014) but not for male infants (p = 0.56).

Bacterial composition in frozen and fresh analysis was measured by the total number of distinct OTUs detected from NP swabs before and after freezing, which ranged from 0 to 15 OTUs per sample. The mean number of OTUs was 6.3 per sample for analysis post freezing and 9.0 per sample for fresh analysis. Results from a random effects model showed that there was a significant association between subjects and the effect of frozen storage on composition (p = 0.0083). The model also indicated significant interaction between the sex of the subject and the effect of freezing on composition. Before freezing the mean number of OTUs for males and females were quite close, 9.5 and 8.7 respectively, however after freezing these were 10.7 and 3.1 respectively, see Figure 1. The difference in composition pre and post freezing was significant for female (p = 0.0014) but not for male infants (p = 0.56).

The effect of freezing NP swabs on the odds of detecting bacterial OTUs was analysed using a random effects logistic regression model, adjusting for sex. The odds of detecting most OTUs did not change with freezing; however, the detection of four taxa was significantly reduced at the 5% level by freezing, see Table 2. Bioinformatics analysis of these T-RFs based on fragment size matched them with specific bacterial taxa as follows; Firmicutes (91 bp), *Pseudomonas sp *(309 bp), Moraxellaceae (147 bp), and *Haemophilus sp/Burkholderia sp*. (518 bp) (see Table 1). After correcting p-values for multiple testing using Holm's method none remained significant, thus the results should be interpreted with caution.

**Table 2:** Effect of frozen storage on the detection of bacterial OTUs by 16S rRNA-based T-RFLP

OTU (bp)	Log odds of detection	p value	95% CI	Closest Bacterial Match (>97% Sequence Similarity)
87	1.81	0.25	-1.25, 4.86	
**91**	**-3.04**	**0.01**	**-5.43, -0.65**	Firmicutes
**114**	**-2.00**	**0.04**	**-3.95, -0.05**	*Moraxella sp.*
116	2.63*	0.36	-2.96, 8.22	*Marinomonas sp.*
133, 215	2.63*	0.36	-2.96, 8.22	
155	1.34	0.20	-0.69, 3.38	
216	-1.18	0.15	-2.80, 0.43	*Corynebacterium propinquum, Rothia mucilaginosa*
273	-2.44	0.26	-6.66, 1.79	Flavobacteriaceae
294	0.94*	0.52	-1.89, 3.76	
**309**	**-2.77**	**0.03**	**-5.34, -0.21**	*Pseudomonas sp.*
392	-2.21	0.06	-4.52, 0.11	
492	-0.67	0.57	-2.99, 1.66	*Clostridiales Incertae Sedis XI*
502	2.63*	0.36	-2.96, 8.22	
504	-2.39	0.25	-6.49, 1.70	*Haemophilus influenzae*
**518**	**-2.59**	**0.01**	**-4.58, -0.61**	*Haemophilus influenzae, Burkholderia fungorum, Comamonadaceae*
520	-0.36	0.68	-2.02, 1.31	*Staphylococcus sp.*
521	-1.70	0.11	-3.80, 0.40	

A random effects logistic regression model adjusting for sex was used. * Indicates OTUs for which sex adjustment was not done. Bold indicates significant results at the 5% level after correcting for multiple testing using Holm's method none of them appeared significant

**Figure 2:**
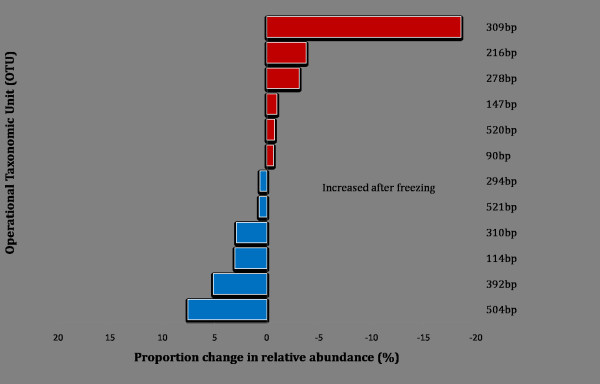
**Bar graph showing proportion change in relative abundance of bacterial OTUs after frozen storage of NP swabs at -70°C analysed by 16S rRNA T-RFLP**. The difference in relative abundance before and after frozen storage was expressed as a proportion of the relative abundance for direct analysis. Most of the 42 OTUs detected showed minimal (<0.5%) change and OTUs that had proportion change >0.5% are shown here.

**Figure 3:**
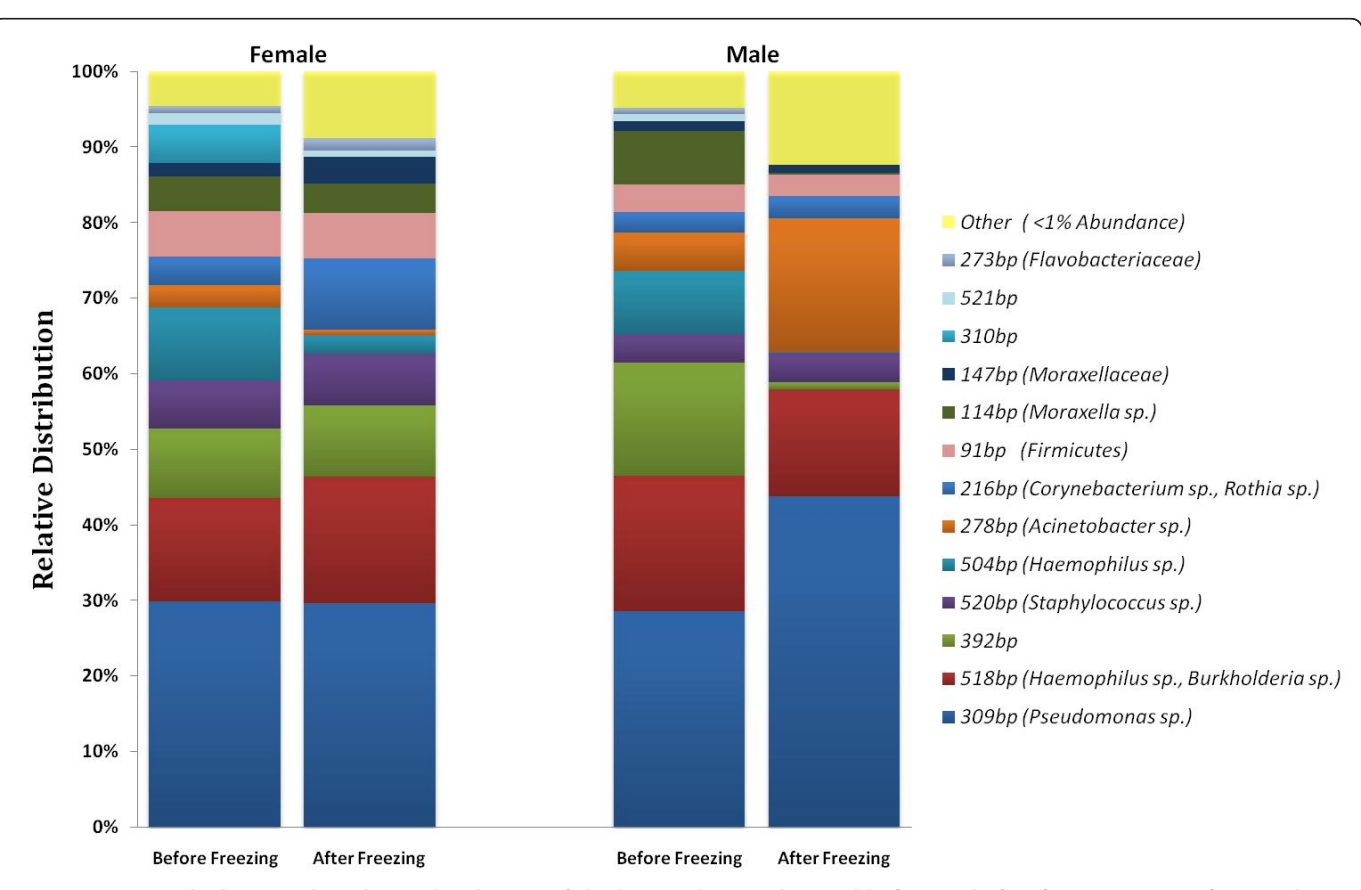
**Bar Graph showing the relative distribution of the bacterial OTUs detected before and after frozen storage of NP swabs at -70°C amongst male and female infants by 16S rRNA-based T-RFLP**. Partial 16S rRNA gene sequences from infant nasopharyngeal clone libraries were BLASTED to identify the microbes (>97% sequence similarity) and *in silico *T-RFLP analysis was used to match them to the OTUs.

The effect of freezing on the relative abundance of OTUs was investigated. For more than 70% of the OTUs, there was less than a 0.5% change in relative abundance after freezing, see Figure 2. 28% of the OTUs had 0.6% to 18.6% shifts in relative abundance post frozen storage, with half showing an increase and the other half showing a decrease in relative abundance. The relative distributions of OTUs were compared for male and female infants as well as before and after freezing, see Figure 3. The relative distributions of bacterial taxa were comparable between male and female infants as well as before and after freezing. The relative proportions of some major taxonomic groups including *Haemophilus sp., Staphylococcus sp. Moraxella sp., *and Firmicutes were comparable before and after freezing for both sexes, see Figure 3. However, the relative proportions of some OTUs including the OTU 392 bp, the 309 bp OTU *(Pseudomonas sp), *the 216 bp OTU (*Rothia sp), *the 278 bp OTU (*Acinetobacter sp.*) and OTUs with relative abundance less than 1% showed some change before and after freezing amongst the infants.

## Discussion

We report preliminary data showing that deep freezing NP swabs in STGG medium at -70°C may have a modest effect on the fingerprint of bacterial communities and a differential effect on the detection of bacterial OTUs found in the infant nasopharynx. Although the relative proportions of some OTUs changed, overall, the relative distributions were comparable before and after frozen storage (Figure 3). Previous studies which investigated the effects of frozen storage on the detection of various microbes in biological samples have shown minimal or no significant effects [1,21,27-29]. Abdullahi *et al.* (2007) reported that the recovery of *S. pneumoniae *from fresh and frozen NP swabs in STGG was consistent, but there were differences in the serotype distributions [21]. This could be attributed to a differential capacity to survive the freezing process among *S. pneumoniae *serotypes. In another culture-based study, there was no effect of freezing on the recovery of *S. pneumoniae *and *S. aureus *from milk samples frozen at -20°C [27,28], but there was an increase in the detection of coagulase negative staphylococci and a decrease in the recovery of *Escherichia coli *and *Actinomyces pyogenes *[28]. Likewise, a recent 16S rRNA gene-based study of Black Band Disease showed that frozen storage increased the proportion of Proteobacteria phylotypes while direct analysis promoted the detection of cyanobacterial and sulfur-oxidizing bacteria [22].

These reports suggest that there may be differential survival capacities to frozen storage among bacterial taxa from the same community. As such, the deep freezing and thawing processes may alter the odds of detection and relative abundance of some, but not all OTUs in a biological sample. In this study, frozen storage significantly altered the odds of detecting a small proportion (<10%) of the bacterial OTUs found (Table 1) and the relative abundance of a couple of OTUs changed by more than 5% after frozen storage (Figure 2). This could be explained by DNA degradation amongst some bacterial taxa; Suomalainen and colleagues demonstrated that the freezing process results in the disintegration of the *Flavobacterium columnare *cell wall, associated with the release of large quantities of DNAse, lysases and proteases[4]. There is further evidence that the structure and stability of bacterial cells influence cryo-preservation of nucleic acids [30].

Interestingly, the observed decrease in the number of OTUs found after frozen storage was significant among female (p = 0.0014) but not among male infants (p = 0.56) (Figure 1). This preliminary data suggests that an effect of freezing on microbial detection may also be differential across sexes. Interestingly, several OTUs detected in both sexes in fresh NP swabs were not detected or were detected at much lower frequencies among female infants post frozen storage. Although sex has been shown to be an important factor in colonization by various bacterial pathogens [31,32], this finding has not been reported elsewhere to our knowledge. It is unclear how an effect of deep freezing on bacterial detection could be differential depending on the sex of a subject, which necessitates further investigations.

Frozen storage of biological samples is necessary for archiving and often done for logistic purposes where real-time processing of samples is not practicable. With the widespread use of vaccines targeting commensals of the respiratory mucosae, it is essential to effectively monitor non-vaccine serotypes and species replacement disease [33,34]. Tracking intra-species serotype replacement and/or switching has overshadowed comprehensive research into the long term effects of vaccination on the microbiome, which may influence health, predisposition to disease and the pathogenesis of various infections [35,36]. Loss or increased detection of particular taxa due to deep frozen storage may have a bearing on microbial ecology and species replacement surveillance. Furthermore, the intricate competitive relations between bacterial phylotypes may not be fully understood if deep freezing alters the fingerprint of microbial communities, albeit modestly [7-10].

In this study, we investigated the effect of frozen storage on NP swabs stored in STGG from 12 infants. However, the sample size and study design limit the validity of the findings. Broad investigations of different biological specimens, storage media, storage duration and microbial detection tools are needed to validate these findings. An effect of frozen storage on microbial detection using culture-based and culture-independent approaches needs to be studied. Finally, further investigation is needed to determine the precise mechanisms by which shifts in microbial community structure occur following frozen storage.

## Conclusions

The potential effects of frozen storage on the composition and relative abundance of microbial populations should not be overlooked with the widespread use of molecular applications in microbial ecology studies.

## Competing interests

The authors declare that they have no competing interests.

## Authors' contributions

BK, MB, RAA and MA proposed the concept and design of this study. BK prepared the clinical samples and performed all the experiments with MA. BK, NIM, DJ and MA performed the data analysis. BK and MA wrote the manuscript with input from all. All authors contributed in discussions and approved the final manuscript.

## Pre-publication history

The pre-publication history for this paper can be accessed here:

http://www.biomedcentral.com/1472-6890/11/2/prepub
